# PSEN2 Mutation Spectrum and Novel Functionally Validated Mutations in Alzheimer’s Disease: Data from PUMCH Dementia Cohort

**DOI:** 10.3233/JAD-220194

**Published:** 2022-06-14

**Authors:** Liling Dong, Caiyan Liu, Longze Sha, Chenhui Mao, Jie Li, Xinying Huang, Jie Wang, Shanshan Chu, Bin Peng, Liying Cui, Qi Xu, Jing Gao

**Affiliations:** a Neurology Department, State Key Laboratory of Complex Severe and Rare Diseases, Peking Union Medical College Hospital, Chinese Academy of Medical Sciences and Peking Union Medical College, Beijing, China; b Institute of Basic Medical Sciences, Peking Union Medical College, Beijing, China

**Keywords:** Alzheimer’s disease, pathogenic mutations, *PSEN2*

## Abstract

**Background:**

The established causative mutations in the *APP*, *PSEN1*, and *PSEN2* can explain less than 1%,Alzheimer’s disease (AD) patients. Of the identified variants, the *PSEN2* mutations are even less common.

**Objective:**

With the genetic study from the dementia cohort of Peking Union Medical College Hospital (PUMCH), we aim to illustrate the *PSEN2* mutation spectrum and novel functionally validated mutations in Chinese AD patients.

**Methods:**

702 AD participants, aged 30–85, were identified in PUMCH dementia cohort. They all received history inquiry, physical examination, biochemical test, cognitive evaluation, brain CT/MRI, and next-generation DNA sequencing. Functional analysis was achieved by transfection of the HEK293 cells with plasmids harboring the wild-type *PSEN2* or candidate mutations.

**Results:**

Nine *PSEN2* rare variants were found, including two reported (M239T, R62C) and seven novel variants (N141S, I368F, L396I, G117X, I146T, S147N, H220Y). The HEK293 cells transfected with the *PSEN2* N141S, M239T, I368F plasmids showed higher Aβ_42_ and Aβ_42_/Aβ_40_ levels relative to the wild-type *PSEN2*. The *PSEN2* L396I, G117X, S147N, H220Y, and R62C did not alter Aβ_42_, Aβ_40_ levels, or Aβ_42_/Aβ_40_ ratio. 1.9%,(13/702) subjects harbored rare *PSEN2* variants. 0.4%,(3/702) subjects carried pathogenic/likely pathogenic *PSEN2* mutations. The three subjects with the functionally validated *PSEN2* mutations were all familial early-onset AD patients. The common symptoms included amnesia and mental symptom. Additionally, the M239T mutation carrier presented with dressing apraxia, visuospatial agraphia, dyscalculia and visual mislocalization.

**Conclusion:**

The *PSEN2* N141S, M239T, and I368F are functionally validated mutations.

## INTRODUCTION

Alzheimer’s disease (AD) is the most common neurodegenerative dementia. The well-established causative genes include amyloid protein precursor (*APP*), presenilin-1 (*PSEN1*), and presenilin-2 (*PSEN2*). They are related to the autosomal dominant form of AD. Based on the previous research, almost 73 *APP*, 322 *PSEN1*, and 87 *PSEN2* variants have been identified (https://www.alzforum.org/). However, they can explain less than 1%,AD population [1]. In this case, many studies are devoted to exploring new causative genes or loci.

The *PSEN2* gene encodes the *PSEN2* protein. It has nine transmembrane domains, a cytoplasmic N-terminus and C-terminus, as well as a cytoplasmic loop between the 6^th^ and 7^th^ transmembrane domains (https://www.alzforum.org/). It is a major component of the γ-secretase, which accounts for the proteolytic cleavage of the *APP* and the formation of amyloid-β (Aβ) peptides [2]. The *PSEN2* and *PSEN1* proteins are highly homologous and share about 60%,amino acid identity [3]. However, of the identified variants, the *PSEN2* mutations are far less than the *PSEN1* mutations. Moreover, most of these *PSEN2* mutations do not have functional evidence.

In this paper, we will illustrate the mutation spectrum of the *PSEN2* gene in the dementia cohort of Peking Union Medical College Hospital (PUMCH). As well, we will disclose the novel functionally validated *PSEN2* mutations in Chinese AD patients.

## METHODS

### Participants

702 participants, aged 30–85, were extracted from PUMCH dementia cohort. They all met the diagnostic criteria for probable AD according to the 2011 recommendations from National Institute on Aging and the Alzheimer’s Association [4]. They all underwent history inquiry, physical examination, biochemical test, cognitive evaluation, and brain CT/MRI. 558 subjects had whole exon sequencing, and 144 cases had targeted exon sequencing of 278 dementia-related genes. This study was approved by the local ethics committee of PUMCH (No. JS-1836). Written informed consent was obtained from the participants.

### Cognitive assessment

Cognitive assessment included neuropsychological screening tests and multi-domain assessment. The former involved Clinical Dementia Rating, Mini-Mental State Exam (MMSE), Activities of Daily Living (ADL) [5, 6], etc. The latter covered executive, visuospatial, linguistic, memory, and reasoning domains. It consisted of Auditory Verbal Learning Test (AVLT), Rey complex figure, word fluency, Digital Symbol Substitution Test, Trail Making Test part A, graphics copying, block design, episodic memory, paired associate learning, similarity, calculation, as well as oral comprehension, repetition, naming, reading, copying, dictation, spontaneous speech and writing, etc. Z-score = (individual score - average score) / standard deviation.

### Gene sequencing

Peripheral blood was collected. Genomic DNA was extracted by QIAamp DNA Blood Mini kit (Qiagen, Hilden, Germany). The DNA libraries were sequenced on NextSeq500 sequencer (Illumina, San Diego, USA). All reads were aligned to human genome reference (UCSC hg19) with Burrows-Wheeler Aligner (version 0.5.9) [7]. The reads were realigned and recalibrated by GATK Indel Realigner (version 3.5) and Base Recalibrator (version 3.5). SNVs and small indels were determined with GATK Unified Genotyper (version 3.5). Variant annotation was performed by Annovar (version 2016Feb01) [8]. The pathogenicity of rare variants was illustrated according to the standards of American College of Medical Genetics and Genomics (ACMG) [9]. The potential pathogenic mutations were validated by Sanger sequencing.

### Functional analysis

The high-purity, endotoxin-free plasmids were prepared by Escherichia coli. HEK293 cells containing Swedish mutant *APP* (*APP*sw) were transfected with plasmids harboring wild-type *PSEN2* (*PSEN2*wt) and candidate *PSEN2* mutations, respectively. The supernatant of cell culture medium was collected after 48 h of transfection. Aβ_40_ and Aβ_42_ levels in the supernatant were determined by ultra-sensitive Aβ_40_ and Aβ_42_ Human ELISA Kit (Thermo, KHB3441, KHB3544). Aβ_40_ and Aβ_42_ values, as well as Aβ_42_/Aβ_40_ ratio were compared between the wild-type and candidate *PSEN2* mutations by Student’s *t* test.

## RESULTS

### Demographic feature and mutation spectrum of AD cohort

As shown in Table 1, 37.9%,(266/702) subjects were males, and 62.1%,(436/702) were females. The age ranged between 41 and 84, with an average of 67.2±9.8 years. Based on the age of onset (AOO), 52.3%,(367/702) subjects were early-onset (AOO < 65 years), while 47.7%,(335/702) were late-onset (AOO≥65 years). 43.6%,(306/702) subjects had at least one first-degree or second-degree relative suffering from dementia, defined as a positive family history of dementia. The *APOE* genotype distribution was as follows: ɛ2/ɛ2 (*n* = 5, 0.7%,), ɛ2/ɛ3 (*n* = 52, 7.4%,), ɛ3/ɛ3 (*n* = 344, 49.0%,), ɛ2/ɛ4 (*n* = 17, 2.4%,), ɛ3/ɛ4 (*n* = 227, 32.3%,), ɛ4/ɛ4 (*n* = 57, 8.1%,).

**Table 1:** Demographic features of 702 AD patients, nine *PSEN2* rare mutation carriers, and three *PSEN2* PLP mutation carriers

	AD (*n* = 702)	*PSEN2* rare mutation carriers (*n* = 9)	*PSEN2* PLP mutation carriers (*n* = 3)
Male/Female *n* (%,)	266 (37.9%,) / 436 (62.1%,)	5 (55.6%,) / 4 (44.4%,)	1 (33.3%,) / 2 (66.7%,)
Age (y)	67.2±9.8	63.7±8.8	54.0±6.0
Disease course (y)	3.2±2.2	3.2±2.0	1.7±0.6
AOO (y)	64.1±9.9	60.4±8.2	52.3±5.5
Early-onset/Late-onset *n* (%,)	367 (52.3%,) / 335 (47.7%,)	6 (66.7%,) / 3 (33.3%,)	3 (100.0%,) / 0 (0.0%,)
FHD+/–*n* (%,)	306 (43.6%,) / 396 (56.4%,)	8 (88.9%,) / 1 (11.1%,)	3 (100.0%,) / 0 (0.0%,)
*APOE* ɛ4 + allele frequency (%,)	25.5%,	33.3%,	16.7%,

AOO, age of onset; FHD, family history of dementia; APOE, apolipoprotein E; AD, Alzheimer’s disease; PLP mutation, pathogenic/likely pathogenic mutations according to the ACMG criteria.

4.0%,(28/702) subjects harbored rare variants in *APP* (*n* = 7), *PSEN1* (*n* = 12), and *PSEN2* (*n* = 9). According to the ACMG criteria, 14 cases carried pathogenic/likely pathogenic (PLP) variants, and the other 14 cases had variant of uncertain significance (VUS). This paper focused on the *PSEN2* mutation spectrum.

### PSEN2 mutation interpretation

As shown in Supplementary Table 1, nine *PSEN2* rare variants were found in the cohort. The *PSEN2* R62C was a published variant [10]. The *PSEN2* M239T was reported by our group in 2021 [11]. The other seven variants were novel, including N141S, I368F, L396I, G117X, I146T, S147N, and H220Y. Of them, eight variants were missense, and one is stopgain. The variants were rare or missing in ExAC, 1000 Genome, Cosmic, or GnomAD databases. They were supposed to be damaging according to SIFT, Polyphen, Mutationtaster, or LRT predictions.

**Fig. 1:**
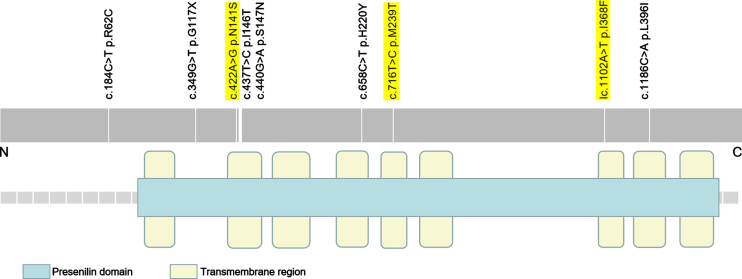
Mutation distribution of *PSEN2* (NM_000447.2). It illustrates the nine *PSEN2* rare variants in this study. The pathogenic/likely pathogenic variants are highlighted in yellow.

As illustrated in Fig. 1, all the variants were within the Presenilin domain except for the *PSEN2* R62C mutation. Moreover, seven variants were located in trans-membrane regions, including the *PSEN2* N141S, M239T, I368F, L396I, I146T, S147N, and H220Y. The *PSEN2* R62C was in intracellular domain adjacent to N-terminus. The G117X was in the extracellular domain between 1st and 2nd trans-membrane regions.

**Fig. 2:**
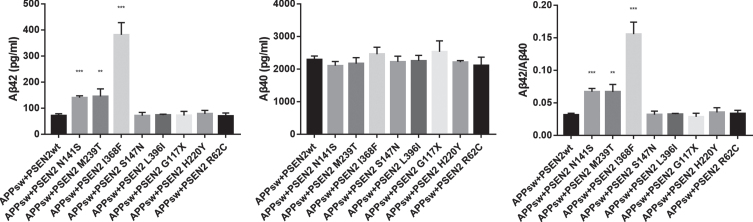
Comparison of Aβ_42_, Aβ_40_ levels, and Aβ_42_/Aβ_40_ ratio between HEK293 cells transfected with wild-type *PSEN2* (*APP*sw+*PSEN2*wt) and candidate *PSEN2* mutations. ^**^p < 0.01, ^***^p < 0.001.

Functional analysis was performed in eight variants. As illustrated in Fig. 2, the HEK293 cells transfected with the *PSEN2* N141S, M239T, I368F plasmids showed increased Aβ_42_ levels (pg/ml) (140.34±7.02, 144.72±29.06, 380.94±47.11 versus 71.54±6.87; *p* < 0.001, *p* = 0.003, *p* = 0.001), as well as elevated Aβ_42_/Aβ_40_ ratios (0.067±0.005, 0.067±0.012, 0.156±0.018 versus 0.031±0.003; *p* < 0.001, *p* = 0.007, *p* = 0.001) relative to the wild-type *PSEN2*. Aβ_40_ levels did not differ between the wild-type *PSEN2* and the *PSEN2* N141S, M239T, and I368F. The other five variants (L396I, G117X, S147N, H220Y, R62C) did not alter Aβ_42_, Aβ_40_ level, or Aβ_42_/Aβ_40_ ratio.

The causative mutations at the residue 141 and 239 of *PSEN2* had been previously confirmed, such as the *PSEN2* N141I and M239V [12, 13]. However, the causative variants at the residue 368 of *PSEN2* had never been reported before. According to the ACMG criteria, the *PSEN2* N141S and M239T were pathogenic, the I368F was likely pathogenic, whereas the other six variants were VUS.

### PSEN2 mutation frequency

1.3%,(9/702) subjects harbored the rare variants in *PSEN2*. 0.4%,(3/702) subjects carried the *PSEN2* PLP mutations. The incidence of the *PSEN2* PLP mutations was higher in the familial and early-onset subjects relative to the sporadic and late-onset patients (1.0%, 0.8%,versus 0%, 0%,).

### Clinical characteristics of PSEN2 PLP mutation carriers

#### Case 1 with PSEN2 p.N141S (c.422A > G)

**Fig. 3:**
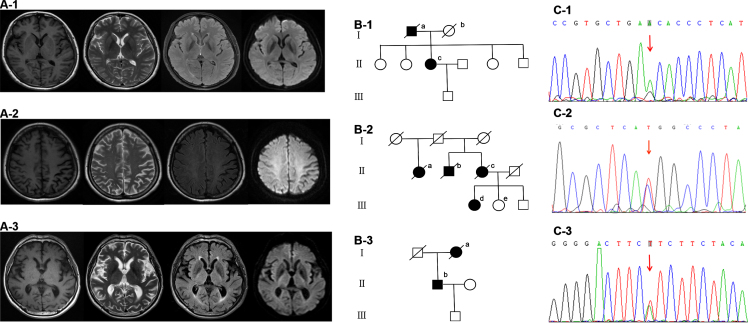
Brain MRI (A1-3), family diagram (B1-3) and Sanger validation of next-Generation sequencing variants (C1-3) in the three subjects harboring functionally validated *PSEN2* pathogenic mutations. A-1, B-1, and C-1 are from case 1 with *PSEN2* p.N141S (c.422A > G). Brain MRI is almost normal. On family diagram (B-1), II-c is the index patient. Her father (I-a) started with memory deficit in his 60s and died about a decade later. Her mother (I-b) died in her 70s without cognitive decline. Her three sisters and one brother are all cognitively normal. A-2, B-2, and C-2 are from case 2 with *PSEN2* p.M239T (c.716T > C). Brain MRI shows left predominant parietal atrophy. On family diagram (B-2), III-d is the index patient. Her 46-year-old sister (III-e) and 39-year-old brother are cognitively normal. Her cousins are all over 50 years old without cognitive impairment. III-e receives gene sequencing which shows no *PSEN2* p.M239T (c.716T > C) mutation. Her mother (II-c), uncle (II-b) and aunt (II-a) all showed cognitive deficit in their 70s and died around 80. The first generation had no cognitive decline when they were alive. However, their age of death was not clear. A-3, B-3, and C-3 are from case 3 with *PSEN2* p.I368F (c.1102A > T). Brain MRI is almost normal. On family diagram (B-3), II-b is the index patient. Her mother (I-a) started with memory impairment in her 70s and died two years later.

The 54-year-old female used to take good care of her family. She always danced with her friends in the community. Things had changed since two years ago. She frequently quarreled with her husband, forgot to use salt when cooking, and asked the same questions repeatedly. She had difficulty in learning a new dance. Her father started with memory deficit in his 60s and died about a decade later. Her mother died in her 70s without cognitive decline. Her siblings were all cognitively normal (Fig. 3).

On admission, she did not think she had any problems. She scored a 28 on the MMSE, and 22 on the ADL. She showed relatively intact performance on memory tests. The z-scores of immediate, short, and long delayed recall of AVLT, recall of complex Rey figure were –0.84, –0.81, –0.52, and –0.3, respectively. Physical examination, electroencephalography, and brain MRI were almost normal (Fig. 3).

Five years later, she was unable to recognize her seven-year-old granddaughter sometimes. She could not remember when her father died or when her son married. She insisted that her husband stole her jewelry and cheated on her. She scored 20 on the MMSE, and 23 on the ADL. On domain assessment, she showed severe deficit on verbal and non-verbal memory tests. The z-scores of long delayed recall of AVLT, paired associate learning and recall of Rey complex figure were –3.38, –3.52, and –4.64, respectively. Executive, visuospatial, reasoning, and linguistic domains were relatively preserved.

#### Case 2 with PSEN2 p.M239T (c.716T > C)

Over the past one year, the 48-year-old right-handed female had encountered difficulty in dressing, writing, and calculating. She always forgot to take medicine. She was restless and irritable. She could do the chores, but very slowly. During sleep she had frequent arousals and sleep-talking.

On admission, she got a 20 on the MMSE and 30 on the ADL. She showed slight deficits on memory domain. The z-scores of short and long delayed recall of AVLT, paired associate learning were –1.8, –2.4, –1.8, respectively. In addition, she showed severe deficit on visuospatial and executive domains. She was unable to finish graphics copying, block design, digital symbol substitution task, and trail making test. She had difficulty in visual and auditory calculation. She spoke fluently with relatively intact naming, repetition, oral and reading comprehension. Writing disturbance was mainly manifested as character structural abnormality. It did not improve with copying the written text. Additionally, she exhibited prominent visual mislocalization. She could answer what animal toys were on the table, and which was the closest to “monkey”. However, when asked to touch “monkey”, she showed great difficulty and always reach in the wrong direction.

Other physical examinations were almost normal, including visual field, acuity, and fundus, etc. Brain MRI showed left predominant parietal atrophy (Fig. 3). The cerebrospinal fluid (CSF) biomarker was in favor of AD diagnosis (Aβ_42_ 439 pg/ml, phosphorylated tau-181 66 pg/ml, total tau 242 pg/ml). Brain ^18^F-AV45 PET showed increased uptake in diffuse cortical regions. Four years later, she could not do any chores. She developed bradykinesia and rigidity of right upper limb and the trunk. She scored 16 on the MMSE, and 53 on the ADL.

On family diagram (Fig. 3), her mother, uncle, and aunt all showed cognitive deficit in their 70s and died around 80. Her siblings and cousins were all cognitively normal. Her 46-year-old sister received gene sequencing which showed no PSEN2 p.M239T (c.716T > C) mutation.

#### Case 3 with PSEN2 p.I368F (c.1102A > T)

This 60-year-old male started with memory impairment two years ago. He could not find his own belongings. So he always suspected his family of stealing from him. He was unable to pick up his grandchild from school as usual since he could not remember the route. He often got lost in the subway station and asked the police to send him home. Occasionally he got up at midnight to brush his teeth and tidy up his clothes and shoes. He was irritable and agitated. He showed word-finding difficulty sometimes. His mother had memory impairment in her 70s and died two years later.

On admission, he scored 7 on the MMSE, and 35 on the ADL. He spoke fluently with intact oral comprehension. Physical examination and brain MRI were almost normal (Fig. 3). Unfortunately, he was lost to follow-up.

### Clinical characteristics of PSEN2 VUS carriers

As illustrated in Supplementary Table 2, six subjects harbored the VUS in the *PSEN2*. They were four males and two females. The AOO ranged between 56 and 70 years old. Five were familial cases, while one was sporadic. They all presented with memory deficit and mental symptom during the initial stage. The other initial symptoms included dyscalculia, disinhibition, impaired oral comprehension, etc. Three cases had parietal and/or temporal lobar atrophy. Four cases were accompanied by periventricular and/or subcortical white matter lesions, Fazekas grade 1-2.

The *PSEN2* G117X was found in a 63-year-old male. His father died of hepatic cancer at the age of 50. His father’s two siblings developed cognitive decline in their 70s. He started with memory deficit, lack of initiative, and restlessness at 56. Gradually, he was unable to calculate, write, or dress himself. On admission, he scored 8 on the MMSE and 55 on the ADL. Brain MRI showed bilateral parietal atrophy.

## DISCUSSION

This is a retrospective study from the PUMCH dementia cohort. 0.4%,(3/702) AD patients carry the *PSEN2* PLP variants. In the early-onset and the familial AD patients, the prevalence of *PSEN2* PLP variants are 0.8%,(3/367) and 1.0%,(3/306), respectively. These are close to the previous findings. Brouwers demonstrated that in the early-onset AD patients, the *PSEN2* mutation frequency is about 1%,[14]. According to a Chinese familial AD research, 1.7%,(7/404) pedigrees harbored *PSEN2* mutations [15].

The HEK293 cells transfected with the plasmids containing the *PSEN2* N141S, M239T, and I368F exhibit increased Aβ_42_ and Aβ_42_/Aβ_40_ in comparison to the wild-type *PSEN2*, indicating their contribution to Aβ_42_ production and AD pathogenesis. The causative mutations at the residue 141 and 239 of *PSEN2* have been reported before, including N141I, N141D, N141Y, M239V, and M239I. Most N141I patients have Volga German ancestry, whereas most M239V and M239I cases have Italian heritage [12, 13, 16]. The *PSEN2* N141D, N141Y, and M239V have been described in Chinese pedigrees with early-onset AD [17–19]. However, it is the first time that the causative mutation at the residue 368 of *PSEN2* has been identified in a familial early-onset AD patient.

The three functionally validated mutations are within the second, fifth, and seventh trans-membrane regions, respectively. The trans-membrane domains of the *PSEN2* are mutation hotspots. The previously reported *PSEN2* p.S175C (c.524C > G), p.Y231C (c.692A > G), and p.T430M (c.1289C > T) are located in the third, fifth, and ninth trans-membrane regions, respectively [20–22].

The three subjects harboring the functionally validated *PSEN2* mutations are all familial early-onset AD patients. They all presented with memory decline and emotional symptom. Additionally, each case had his own characteristic manifestations. The subject with the *PSEN2* N141S mutation showed prominent mental symptom with lack of insight. She denied that she has any problems and refused to see a doctor. Unlike the *PSEN2* N141S carrier, the subject harboring the *PSEN2* M239T mutation complained of her “locked” brain and had a strong willingness to seek medical treatment. She also manifested with dressing apraxia, visuospatial agraphia, dyscalculia, visual mislocalization, and sleep disorder. The subject with the *PSEN2* I368F mutation was characterized by spatial and temporal disorientation, as well as word-finding difficulty.

The *PSEN2* N141S and I368F mutations have not been reported before. Our group reported the *PSEN2* M239T mutation in 2021 [11]. Li et al. reported another Chinese early-onset AD patient harboring the *PSEN2* M239T in 2021. He presented with visuospatial and memory impairment [23].

It is the first time that visual mislocalization has been described in an AD patient with the *PSEN2* mutation. It is a disorder of visually guided reaching, which might be due to the left prominent parietal involvement in case 2. As illustrated by Husain et al., the parietal cortex receives sensory inputs, such as visual, somatosensory, and vestibular stimuli [24]. Meanwhile, it has reciprocal connections to premotor cortex, hippocampus, etc. It plays a crucial role in the perception and integration of sensory information, as well as in the association of sensory and motor signals, directing attention, especially for localizing objects at different spatial locations [24].

Among the three cases, the subject harboring the *PSEN2* I368F mutation showed the worst cognitive performance on admission. Among the three mutations, the *PSEN2* I368F mutation had the highest CSF Aβ_42_ level and Aβ_42_/Aβ_40_ ratio in *in vitro* studies. We suppose that the *in vitro* greater Aβ_42_ or Aβ_42_/Aβ_40_ level of the specific mutation might predict the faster disease progression in the subject harboring the mutation. The previous *in vivo* studies showed that the low Aβ_42_ level and high tau/Aβ_42_ ratio correlated with the rapid cognitive decline [25]. The association between the *in vitro* and *in vivo* biomarkers, as well as the disease progression should be further investigated.

In conclusion, we report three functionally validated *PSEN2* mutations, including the *PSEN2* p.N141S (c.422A > G), p.M239T (c.716T > C), and p.I368F (c.1102A > T). The *PSEN2* L396I, G117X, S147N, H220Y, and R62C do not alter Aβ_42_, Aβ_40_ level, or Aβ_42_/Aβ_40_ ratio in *in vitro* transfection studies. Nevertheless, these variants cannot be necessarily determined as non-pathogenic based on these results alone. For instance, the *APP* p. E693G (c.2078A > G) mutation did not increase Aβ_42_, Aβ_40_, or Aβ_42_/Aβ_40_, either. However, it formed protofibrils at a high rate, which contributed to accelerated insoluble Aβ deposits [26]. Therefore, the pathogenicity of these variants needs to be clarified by further functional validation and pathological examination. The main limitation of this study is the absence of CSF biomarkers and pathological evidence. These are expected to confirm the *in vivo* pathological effect of these mutations.

## Supplementary Material

Supplementary Material
