# Supplementary material for: PSEN2 Mutation Spectrum and Novel Functionally Validated Mutations in Alzheimer’s Disease: Data from PUMCH Dementia Cohort
Source: J Alzheimers Dis. 2022 Jun 14;87(4):1549–56. doi: 10.3233/JAD-220194 (PMC9277672; doi:10.3233/JAD-220194)
Supplement: Supplementary Material [file jad-87-jad220194-s001.pdf]

Supplementary Material

PSEN2 Mutation Spectrum and Novel Functionally Validated Mutations in Alzheimer's Disease: Data from PUMCH Dementia Cohort

Supplementary Table 1. Interpretation of nine *PSEN2* rare variants (NM_000447.2)

Variant	1000genome/ExAC/ Cosmic/GnomAD	SIFT/Polyphen/LRT/ Mutationtaster	Domain	Functionally validation	ACMG
c.422A>G p.N141S	-/-/-/-	D/D/D/D	Presenilin domain, 2nd trans-membrane region	+	Pathogenic
c.716T>C p.M239T	-/-/-/-	D/D/D/D	Presenilin domain, 5th trans-membrane region	+	Pathogenic
c.1102A>T p.I368F	-/-/-/-	D/D/D/D	Presenilin domain, 7th trans-membrane region	+	Likely pathogenic
c.184C>T p.R62C	-/-/-/0.0002	D/D/N/N	Intracellular domain adjacent to N-terminus	-	VUS
c.349G>T p.G117X	-/-/-/-	-/-/N/A	Presenilin domain, extracellular domain between 1st and 2nd trans-membrane regions	-	VUS
c.1186C>A p.L396I	-/-/-/-	D/D/D/D	Presenilin domain, 8th trans-membrane region	-	VUS
c.437T>C p.I146T	-/-/-/0.000004	D/P/D/D	Presenilin domain, 2nd trans-membrane region	NA	VUS
c.440G>A p.S147N	-/-/-/-	D/P/D/D	Presenilin domain, 2nd trans-membrane region	-	VUS
c.658C>T p.H220Y	-/-/-/-	D/D/D/D	Presenilin domain, 4th trans-membrane region	-	VUS

VUS, variant of uncertain significance; NA, not applicable.

Supplementary Table 2. Clinical data of nine *PSEN2* mutation carriers

Case	Variant	Gender	Age/ AOO	FHD	Education (y)	Handed ness	*APOE*	MMSE/ ADL	Clinical symptom in the early stage	Brain MRI
1	N141S	Female	54/52	+	12	Right	ε3ε4	28/22	Memory deficit, mental symptom, lack of insight	Normal
2	M239T	Female	48/47	+	9	Right	ε3ε3	20/30	Memory deficit, mental symptom, dressing apraxia, visuospatial agraphia, dyscalculia, visual mislocalization, sleep disorder	Left predominant parietal atrophy
3	I368F	Male	60/58	+	0	Right	ε3ε3	7/35	Memory deficit, mental symptom, spatial and temporal disorientation, word-finding difficulty	Normal
4	R62C	Male	75/70	+	3	Right	ε3ε4	3/68	Memory deficit, mental and psychiatric symptom, dyscalculia, tremor	Bilateral temporal atrophy, periventricular white matter lesions, Fazekas grade 2
5	G117X	Male	63/56	+	8	Right	ε4ε4	8/55	Memory deficit, mental symptom	Bilateral parietal atrophy
6	L396I	Male	68/63	+	8	Right	ε2ε4	15/40	Memory deficit, mental and psychiatric symptom, disinhibition	Periventricular white matter lesions, Fazekas grade 1-2
7	I146T	Female	72/70	+	9	Right	ε3ε4	8/38	Memory deficit, mental and psychiatric symptom, impaired oral comprehension, bradykinesia	Bilateral tempo-parietal atrophy, periventricular and subcortical white matter lesions, Fazekas grade 2
8	S147N	Male	62/59	+	0	Right	ε3ε3	16/37	Memory deficit, mental symptom	Normal
9	H220Y	Female	71/69	-	9	Right	ε3ε3	13/35	Memory deficit, mental symptom	Periventricular and subcortical white matter lesions, Fazekas grade 2

AOO, age of onset; FHD, family history of dementia; MMSE, Mini-Mental State Exam; ADL, Activities of Daily Living; *APOE*, apolipoprotein E
